# Genome Skimming Contributes to Clarifying Species Limits in *Paris* Section *Axiparis* (Melanthiaceae)

**DOI:** 10.3389/fpls.2022.832034

**Published:** 2022-04-04

**Authors:** Yunheng Ji, Jin Yang, Jacob B. Landis, Shuying Wang, Lei Jin, Pingxuan Xie, Haiyang Liu, Jun-Bo Yang, Ting-Shuang Yi

**Affiliations:** ^1^CAS Key Laboratory for Plant Diversity and Biogeography of East Asia, Kunming Institute of Botany, Chinese Academy of Sciences, Kunming, China; ^2^Yunnan Key Laboratory for Integrative Conservation of Plant Species With Extremely Small Population, Kunming Institute of Botany, Chinese Academy of Sciences, Kunming, China; ^3^School of Life Sciences, Yunnan University, Kunming, China; ^4^Section of Plant Biology and the L. H. Bailey Hortorium, School of Integrative Plant Science, Cornell University, Ithaca, NY, United States; ^5^BTI Computational Biology Center, Boyce Thompson Institute, Ithaca, NY, United States; ^6^School of Traditional Chinese Medicine, Guangdong Pharmaceutical University, Guangzhou, China; ^7^State Key Laboratory of Phytochemistry and Plant Resources in West China, Kunming Institute of Botany, Chinese Academy of Sciences, Kunming, China; ^8^Germplasm Bank of Wild Species, Kunming Institute of Botany, Chinese Academy of Sciences, Kunming, China

**Keywords:** plastome, ribosomal DNA, taxonomy, *Paris* Linn., species delimitation

## Abstract

Paris L. section *Axiparis* H. Li (Melanthiaceae) is a taxonomically perplexing taxon with considerable confusion regarding species delimitation. Based on the analyses of morphology and geographic distribution of each species currently recognized in the taxon, we propose a revision scheme that reduces the number of species in *P.* sect. *Axiparis* from nine to two. To verify this taxonomic proposal, we employed a genome skimming approach to recover the plastid genomes (plastomes) and nuclear ribosomal DNA (nrDNA) regions of 51 individual plants across the nine described species of *P.* sect. *Axiparis* by sampling multiple accessions per species. The species boundaries within *P.* sect. *Axiparis* were explored using phylogenetic inference and three different sequence-based species delimitation methods (ABGD, mPTP, and SDP). The mutually reinforcing results indicate that there are two species-level taxonomic units in *P*. sect. *Axiparis* (*Paris forrestii s.l.* and *P. vaniotii s.l.*) that exhibit morphological uniqueness, non-overlapping distribution, genetic distinctiveness, and potential reproductive isolation, providing strong support to the proposed species delimitation scheme. This study confirms that previous morphology-based taxonomy overemphasized intraspecific and minor morphological differences to delineate species boundaries, therefore resulting in an overestimation of the true species diversity of *P.* sect. *Axiparis*. The findings clarify species limits and will facilitate robust taxonomic revision in *P.* sect. *Axiparis*.

## Introduction

Species delimitation is the crucially important first step for designing research in many fields of biology (Mace, 2004). Traditionally, species boundaries are defined by taxonomists based on the analysis of morphological variation (Lewin, 1981; Henderson, 2005), which has resulted in massive disagreements over species identification and delimitation due to either phenotypic plasticity or lack of taxonomically robust morphological characters at the species level (Hebert et al., 2003; De Queiroz, 2007). To compensate, additional data types, such as molecular and ecological profiles, are needed to explicitly decipher species boundaries (Sites and Crandall, 1997; Sites and Marshall, 2003; Ellis et al., 2006; Bickford et al., 2007; Duminil and Michele, 2009; Su et al., 2015; Eisenring et al., 2016; Lambert et al., 2017; Sukumaran and Knowles, 2017; Cheng et al., 2020; Ma et al., 2020).

Analysis of DNA sequence variation can provide useful information for identifying and delineating species (Brower et al., 1996; Hebert et al., 2003; Kress et al., 2005; Pons et al., 2006; Hollingsworth et al., 2009, 2011, 2016; Hollingsworth, 2011; Duminil et al., 2012; Puillandre et al., 2012). With next-generation DNA sequencing (NGS) technologies, genome-wide sequence variation has begun to replace one or a few sequence loci for the identification and delimitation of plant species (Li et al., 2015; Coissac et al., 2016; Hollingsworth et al., 2016). The genome skimming approach, which uses NGS technologies to generate multi-copy and highly repetitive genome components, such as whole plastid genomes (plastomes) and nuclear ribosomal DNA (nrDNA) clusters *via* relatively low coverage genome sequencing (Straub et al., 2012), has been increasingly used for species identification and delimitation in recent years (Nock et al., 2011; Kane et al., 2012; Dodsworth, 2015; Li et al., 2015; Ruhsam et al., 2015; Firetti et al., 2017; Fu et al., 2019, 2021; Ji et al., 2019a,^2020^, ^2021^; Knope et al., 2020; Ślipiko et al., 2020; Su et al., 2021). Compared with restriction-site associated DNA sequencing (RAD-seq; Miller et al., 2007; Baird et al., 2008), another NSG-based technique that is extensively used to generate genomic data for plant species identification and delimitation (e.g., Wu et al., 2018; Donkpegan et al., 2020; Ma et al., 2020; Zhou et al., 2020; Li et al., 2021), the promising advantage of using genome skimming for species identification and delimitation is the avoidance of problems encountered with RAD-seq (Kane et al., 2012; Hollingsworth et al., 2016), such as only applying to diploids and generating asymmetric data between distinctly related taxa.

**FIGURE 1:**
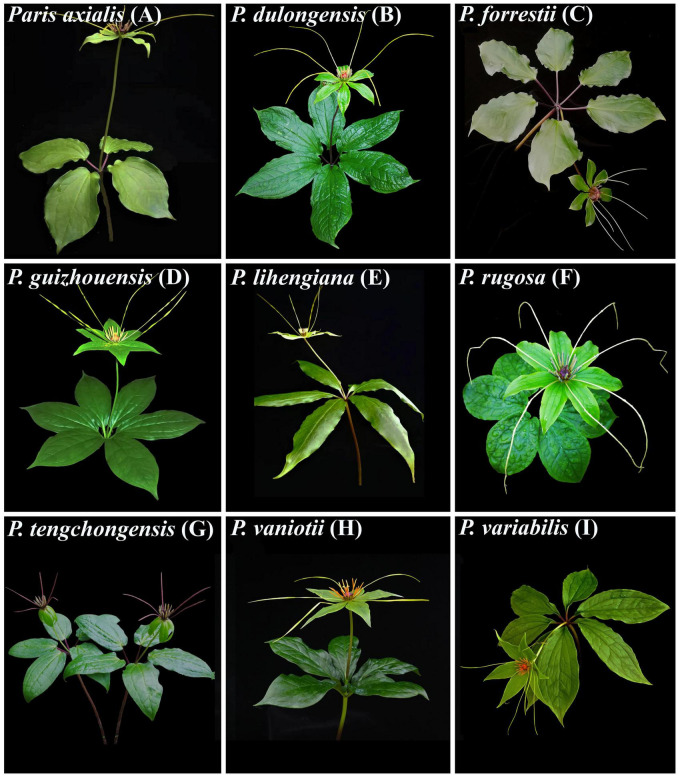
Nine nominal species within *Paris* section *Axiparis*, showing their leaf size and shape variations. *Paris axialis* **(A)**, *P. dulongensis* **(B)**, *P. forrestii* **(C)**, *P. guizhouensis* **(D)**, *P. lihengiana* **(E)**, *P. rugosa* **(F)**, *P. tenchongensis* **(G)**, *P. vaniotii* **(H)**, and *P. variabilis* **(I)**.

**FIGURE 2:**
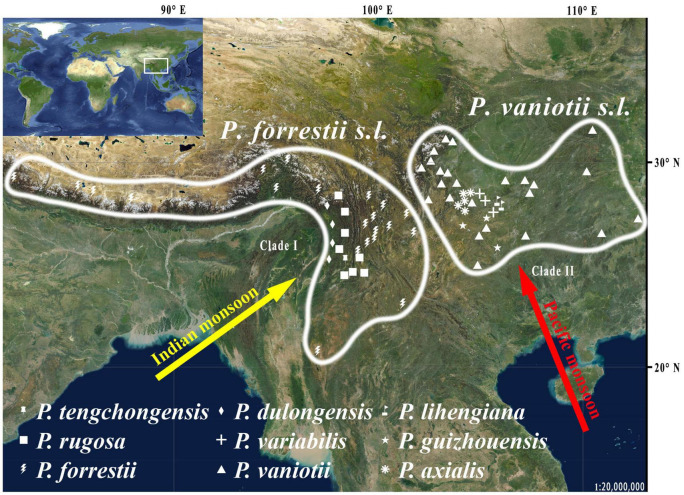
Distribution of the nine nominal species within *Paris* section *Axiparis.*

This study focuses on *Paris* Linn. section *Axiparis* H. Li (Melanthiaceae), a taxonomically perplexing plant group that includes nine described species distributed from Central China to the Himalayas (Li, 1998; Ji et al., 2006, 2019b; Huang et al., 2016). Since the description of the first two species (*Paris vaniotii* and *P. forrestii*) of the section (Léveillé, 1906; Takhtajan, 1983), a total of four (*P. axialis*, *P. guizhouensis*, *P. lihengiana*, and *P. variabilis*) and three (*P. dulongensis*, *P. rugosa*, and *P. tengchongensis*) species whose morphologies are similar to *P. vaniotii* and *P. forrestii* respectively have been described (Li, 1984, 1992; He, 1990; Ji et al., 2017; Xu et al., 2019; Yang et al., 2019). The rapid accumulation in the number of species over the last 40 years has led to considerable taxonomic confusion in *P.* sect. *Axiparis*. After critical examination of types and specimens assigned to these species, it was found that, except for slight differences in leaf shape and size (Figure 1), these recently described species exhibit high levels of similarity in flower, fruit, and seed morphology with *P. vaniotii* and *P. forrestii* (Ji, 2021). Remarkably, leaf shape and size of foliar displays have high levels of intraspecific variation that sometimes far exceeds the divergence used for species diagnosis (Ji, 2021). Also noteworthy, we found that *P. dulongensis*, *P. rugosa*, and *P. tengchongensis* share the morphological similarity of stamens numbering twice as many as the sepal number (two-whorled stamens), and being geographically distributed from the Hengduan Mountains (Southwestern China) to the Himalayas (Figure 2). Comparatively, *P. axialis*, *P. guizhouensis*, *P. lihengiana*, *P. vaniotii*, and *P. variabilis*, have stamens numbering three times that of the sepal number (three-whorled stamen), and these species are distributed from Central China to the Wumeng Mountains in Southwestern China (Figure 2). The overlap of morphological features and species ranges implies that (1) *P. dulongensis*, *P. rugose*, and *P. tengchongensis* are likely conspecific with *P. forrestii*, and (2) *P. axialis*, *P. guizhouensis*, *P. lihengiana*, and *P. variabilis* may belong to *P. vaniotii*. Accordingly, previous morphological-based taxonomic studies (Li, 1984; He, 1990; Li, 1992; Ji et al., 2017; Xu et al., 2019; Yang et al., 2019) may have overemphasized intraspecific and minor morphological variations to establish species, therefore led to the proliferation of synonyms in *P.* sect. *Axiparis*.

Due to the above-mentioned aspects, *P.* sect. *Axiparis* is in great need of taxonomic revision. In this study, we attempt to use genome skimming to establish a basic understanding of species boundaries in this taxonomically perplexing taxon. Specifically, we generated complete plastomes and nrDNA sequences by sampling multiple accessions per species within *P.* sect. *Axiparis via* low coverage genome sequencing. Based on phylogenetic inference and multiple sequence-based species delimitation methods, we aim to test our taxonomic proposal that reduces the number of species in *P.* sect. *Axiparis* from nine to two (*Paris forrestii s.l.* and *P. vaniotii s.l.*).

## Materials and Methods

### Plant Samples, Illumina Sequencing, Assembly, and Annotation

In total, we sampled 51 individual plants including representatives of each of the nine described species of *P.* sect. *Axiparis*, in which 46 accessions were newly sequenced in the present study (Table 1). Voucher specimens and leaf tissue were collected from the field. For each taxon, multiple individuals within a species representing different localities were included, and at least one accession was harvested from the type locality. The sampling strategy entirely covers the nine species’ distribution ranges and allows robust exploring of species-level monophyly and species boundaries within *P.* sect. *Axiparis* to critically test for our taxonomic proposal.

**TABLE 1:** Plant samples used in this study with voucher information and GenBank accession numbers.

Taxa	Locality	Voucher*	GenBank accessions	
			Complete plastome	Ribosomal DNA
*Paris axialis*	Daguan, Yunnan, China	Ji YH 2019049	MW229127	MW202302
*P. axialis*	Shuifu, Yunnan, China	Ji YH and Yang CJ 042	MW229087	MW202314
*P. axialis*	Yilang, Yunnan, China	Ji YH and Yang CJ 047	MW229099	MW202318
*P. axialis*	Zhenxiong, Yunnan, China	Ji YH and Yang CJ 052	MW229086	MW202317
*P. dulongensis*	Gongshan, Yunnan, China	Yang CJ and Zhou GH 001	MW229123	MW202303
*P. dulongensis*	Gongshan, Yunnan, China	Yang CJ and Zhou GH 004	MW229126	MW202313
*P. dulongensis*	Lushui, Yunnan, China	Ji YH 2016521	MW229091	MW202284
*P. dulongensis*	Tengchong, Yunnan, China	Ji YH 2016527	MW229115	MW202285
*P. dulongensis*	Tengchong, Yunnan, China	Ji YH 2016528	MW229103	MW202286
*P. dulongensis*	Tengchong, Yunnan, China	Ji YH 2016529	MW229090	MW202287
*P. dulongensis*	Gongshan, Yunnan, China	Li H and Ji YH 056	MW229094	MW202301
*P. dulongensis*	Gongshan, Yunnan, China	Li H 57**	MN125566	MN174887
*P. forrestii*	Tengchong, Yunnan, China	Wang ZM 001	MW229119	MW202306
*P. forrestii*	Tengchong, Yunnan, China	Wang ZM 002	MW229117	MW202307
*P. forrestii*	Jumla, Nepal	Zhou GH 002	MW229121	MW202308
*P. forrestii*	Chayu, Tibet, China	Yang CJ and Zhou GH 014	MW229124	MW202309
*P. forrestii*	Chayu, Tibet, China	Yang CJ and Zhou GH 015	MW229122	MW202311
*P. forrestii*	Jumla, Nepal	Li JX *s. n.*	MW229125	MW202312
*P. forrestii*	Changning, Yunnnan, China	Ji YH 2016557	MW229111	MW202290
*P. forrestii*	Changning, Yunnnan, China	Ji YH 2016560	MW229088	MW202291
*P. forrestii*	Gongshan, Yunnan, China	Zhou GH s. n.**	MN125565	MN174877
*P. forrestii*	Fugong, Yunnan, China	Li FR *s. n.*	MW229106	MW202293
*P. guizhouensis*	Shuicheng, Guizhou, China	Ji YH 2016032	MW229093	MW202322
*P. guizhouensis*	Daozhen, Guizhou, China	Ji YH 201603901	MW229085	MW202296
*P. guizhouensis*	Dafang, Guizhou, China	Ji YH 2016046	MW229096	MW202297
*P. lihengiana*	Weixin, Yunnan, China	Ji YH 2016052	MW229114	MW202298
*P. lihengiana*	Yanjin, Yunnan, China	Ji YH 2018031	MW229107	MW202324
*P. lihengiana*	Daguan, Yunnan, China	Ji YH 2018041	MW229108	MW202300
*P. rugosa*	Fengqing, Yunnan, China	Ji YH 2016530	MW229082	MW202288
*P. rugosa*	Fengqing, Yunnan, China	Ji YH 2016531	MW229083	MW202289
*P. rugosa*	Changning, Yunnan, China	Ji YH 2016524	MW229084	MW202292
*P. rugosa*	Changning, Yunnan, China	Ji YH 2016525**	MN125570	MN174872
*P. rugosa*	Tengchong, Yunnan, China	Ji YH 2019033	MW229120	MW202304
*P. rugosa*	Tengchong, Yunnan, China	Ji YH 2019034	MW229128	MW202305
*P. rugosa*	Gongshan, Yunnan, China	Ji YH 2019067	MW229118	MW202310
*P. rugosa*	Longyang, Yunnan, China	Yang FJ *s. n.*	MW229092	MW202282
*P. tengchongensis*	Tengchong, Yunnan, China	Ji YH 2017211	MW229098	MW202279
*P. tengchongensis*	Tengchong, Yunnan, China	Ji YH 2017212	MW229089	MW202280
*P. tengchongensis*	Tengchong, Yunnan, China	Ji YH 2016038	MW229095	MW202244
*P. tengchongensis*	Tengchong, Yunnan, China	Ji YH 201603902**	MN125584	MN174889
*P. tengchongensis*	Tengchong, Yunnan, China	Ji YH 2016040	MW229113	MW202278
*P. tengchongensis*	Tengchong, Yunnan, China	Ji YH 2017013	MW229112	MW202281
*P. tengchongensis*	Tengchong, Yunnan, China	Ji YH 2016391	MW229116	MW202283
*P. vaniotii*	Guangyuan, Sichuan, China	Ji YH 2016671	MW229109	MW202299
*P. vaniotii*	Xin Ning, Hunan, China	Li H 052**	MW229102	MN174901
*P. vaniotii*	Nanchan, Chongqing, China	Ji YH 2016693	MW229104	MW202321
*P. vaniotii*	Guanyang, Guangxi, China	Ji YH 2016652	MW229110	MW202295
*P. vaniotii*	Weining, Guizhou, China	Ji YH and Yang CJ 028	MW229097	MW202319
*P. variabilis*	Shuifu, Yunnan, China	Ji YH and Yang CJ 039	MW229105	MW202315
*P. variabilis*	Yanjin, Yunnan, China	Ji YH and Yang CJ 049	MW229100	MW202316
*P. variabilis*	Zhenxiong, Yunnan, China	Ji YH and Qiu Bin 004	MW229101	MW202320

*Voucher specimens are deposited at the Herbarium of Kunming Institute of Botany, Chinese Academy of Sciences (KUN), **The complete plastomes and nrDNA sequences of these samples were sequenced in previous studies (Ji et al., 2019b,^2020^).

Total genomic DNA for each accession was isolated from ∼10 mg silica gel dried leaf tissues using cetyltrimethylammonium bromide (CTAB; Doyle and Doyle, 1987). Approximately 5 μg of purified genomic DNA was used to construct PCR-free shotgun libraries with a TruSeq DNA Sample Prep Kit (Illumina, Inc., San Diego, CA, United States) according to the manufacturer’s instructions. Paired-end (150 bp) sequencing was performed on the Illumina HiSeq 2500 platform (Illumina, Inc., San Diego, CA, United States) to generate approximately two giga base pairs (Gbp) of raw data for each sample. Remarkably, *Paris* is a fairly distinctive angiosperm genus in possessing giant genomes (Pellicer et al., 2014; Ji, 2021), and the minimum documented genome size in the genus (*P. bashanensis*, 1C = 28.73 Gbp; Ji, 2021) is much larger than the mean genome size of angiosperms (1C = 5.7 Gbp; Pellicer et al., 2014). Due to the relatively low sequencing coverage and lack of nuclear genome reference, it is difficult to recover a sufficient number of unlinked and single-copy nuclear loci from the genome skimming data. Therefore, only complete plastomes and nrDNA sequences were assembled in this study for phylogenetic reconstruction and species delimitation analyses.

Shotgun reads of each sample were deposited in NCBI short-read archive (SRA), with accession numbers being shown in Supplementary Table 1. Trimmomatic v0.40 (Bolger et al., 2014) was used to remove low-quality reads and adaptors from the Illumina raw reads with default parameters. The filtered reads were assembled into complete plastomes and nrDNA clusters with the GetOrganelle pipeline v1.7.5.0 (Jin et al., 2020) on a Linux system, using the previously published complete plastome (MN125565) and nrDNA sequence (MN174877) of *P. forrestii* (Ji et al., 2019b) as the reference. The assembled plastomes were annotated using the online software Geseq v2.03 (Tillich et al., 2017), with default parameters. The transfer RNA (tRNA) genes were further verified using tRNAscan-SE v2.0 (Lowe and Chan, 2016) with default parameters. For nrDNA annotation, the ribosomal RNA genes (26S, 18S, and 5.8S ribosomal RNA genes) and boundaries with the intergenic transcribed spacer (ITS) regions were annotated and defined by comparison with the reference in Geneious v10.2.3 (Kearse et al., 2012).

### Data Analyses

The complete plastome and entire nrDNA clusters (including 18S rRNA, ITS1, 5.8S rRNA, ITS2, and 26S rRNA) were aligned using MAFFT v7.450 (Katoh and Standley, 2013). The alignments of sequences were deposited the online database Treebase,^1^ and only single nucleotide polymorphisms (SNPs) were included in phylogenetic and species delimitation analyses. Partition homogeneity tests (Farris et al., 1994) were performed with PAUP* 4.0b10 (Swofford, 2002) to determine the degree of congruence between the concatenated plastome and nrDNA matrices, as well as between coding (18S rRNA, 5.8S rRNA, and 26S rRNA) and non-coding regions (ITS1 and ITS2) of nrDNA sequences, using a heuristic tree search algorithm for 500 replicates.

Phylogenetic relationships were inferred using both maximum likelihood (ML) and Bayesian inference (BI) methods, based on which we tested whether the nine described species within *P.* sect. *Axiparis* are monophyletic units. Previous studies revealed that ancient intergeneric hybridization may have occurred between *Trillium* and *P.* sect. *Paris* (resulting in the speciation of *P. japonica*: *P.* sect. *Kinugasa*), and past intersectional hybridization likely took place between *P.* sect. *Axiparis* and *P.* sect. *Euthyra* (Ji et al., 2019b; Ji, 2021). Given that such reticulate relationships may result in phylogenetic errors (Philippe et al., 2011), we selected *P. thibetica* (complete plastome: MN125569; nrDNA: MN174890; Ji et al., 2019b) as the outgroup, which represents *P.* sect. *Thibeticae*, the closest relative of *P.* sect. *Axiparis* (Ji et al., 2006, 2019b; Ji, 2021). The best-fit sequence substitution model (GTR + G for concatenated plastome matrix, and GTR + I + G for concatenated nrDNA matrix) were determined by Modeltest v3.7 (Posada and Crandall, 1998) using the Akaike information criterion (Posada and Buckley, 2004). The ML analysis was performed with RAxML-HPC BlackBox v8.1.24 (Stamatakis, 2006). The best-scoring ML tree for each dataset was produced with 1,000 bootstrap (BS) replicates to provide support values for each node. The BI tree was inferred using MrBayes v3.2 (Ronquist et al., 2012). Two independent Markov chain Monte Carlo (MCMC) runs were performed with one million generations, sampling every 100 generations, with the initial 25% of the sampled trees as burn-in. Posterior probability (PP) values were computed based on the remaining trees.

Relying on reciprocal monophyly alone to delineate species boundaries likely under-represents true species-level diversity (Ence and Carstens, 2011; Rannala and Yang, 2013). The development of some coalescence-based species delimitation methods, such as Bayesian Phylogenetics and Phylogeography (BPP; Rannala and Yang, 2013), and spedeSTEM (Ence and Carstens, 2011), provide the solution to this issue since these methods do not require reciprocal monophyly of any species in a given gene tree. Both BPP and spedeSTEM are only suitable for processing genomic segments no longer than 500 or 1,000 bp (Ence and Carstens, 2011; Rannala and Yang, 2013), and thus cannot be used to analyze either complete plastomes or entire nrDNA sequences. Accordingly, we used the following approaches to explore species boundaries within *P.* sect. *Axiparis* based on each dataset (concatenated plastome and nrDNA matrices): (1) the distance-based method automatic barcode gap discovery (ABGD; Puillandre et al., 2011), (2) the tree-based method multi-rate Poisson tree processes model (mPTP; Kapli et al., 2017), and (3) the coalescence-based method species delimitation plugin (SDP; Masters et al., 2011) in Geneious v.10.2.3 (Kearse et al., 2012). The outgroup (*P. thibetica*) sequences were removed from the species delimitation analyses.

The ABGD analyses were conducted with the online server^2^ with default settings (Pmin = 0.001, Pmax = 0.1, Steps = 10, *X* = 1.5, Nb = 20). All three genetic distance models (JC69, K2P, and uncorrected *P*-distances) specified by the program were used. Next, we used the mPTP v0.2.3 algorithm (Kapli et al., 2017), an improvement to PTP (Zhang et al., 2013), to delineate species boundaries and to estimate the posterior probability (PP) values for the putative species. The mPTP analyses were performed on the web server^3^ with standard default settings (The MCMC algorithm was conducted for 0.1 million generations, sampling every 100 generations and a 10% burn-in), using the inferred ML trees of plastome and nrDNA as inputs since the branch lengths of ML tree represent expected numbers of substitutions per site. Based on ML and BI trees of the concatenated plastome and nrDNA matrices, we performed SDP analyses to generate species delimitation scheme in *P.* sect. *Axiparis*. The distinctiveness of these candidate species proposed by SDP analyses was estimated using Rosenberg’s *P*_(AB)_: the probability of reciprocal monophyly under a random coalescent model (Rosenberg, 2007), and Rodrigo’s *P*_(_*_*R*_*_*D)*_: the probability that a clade has the observed degree of distinctiveness due to a random coalescent process (Rodrigo et al., 2008).

## Results

### Genome Skimming

The summary of low coverage genome sequencing and assembly of plastome and nrDNA sequences is presented in Supplementary Table 1. Plastome assembly generated the complete plastome of all samples, which possess a typical quadripartite structure, with the sequence length varying from 156,061 to 157,653 bp. The plastomes identically contain 114 genes, including 80 protein-coding genes, 30 tRNA genes, and four plastid rRNA genes (Supplementary Table 2). In addition, the assembly of nrDNA entirely covered 18S, ITS1, 5.8S, ITS2, and 26S regions in all accessions, with the sequence length being either 5,851 or 5,852 bp. DNA sequences of the newly generated plastomes and nrDNA sequences in this study were deposited in GenBank, with accession numbers being shown in Table 1.

### Phylogenetic Inferences

**FIGURE 3:**
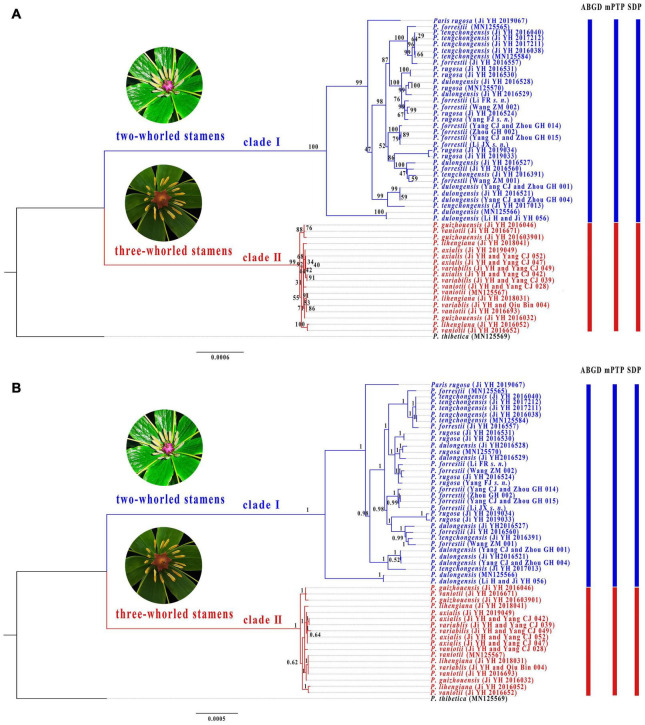
Phylogenetic tree of *Paris* section *Axiparis* based on Maximum likelihood (ML, **A**) and Bayesian inference (BI, **B**) analyses of complete plastomes. Numbers above branches indicate ML bootstrap (BS) percentages and Bayesian posterior probabilities (PP). Species delimitation schemes proposed by automatic barcode gap discovery (ABGD), multi-rate Poisson tree processes model (mPTP), and species delimitation plugin (SDP) of Geneious are reflected on the tree topology.

Alignment of the plastome sequences yielded a matrix of 160,681 positions, in which we identified 1,724 variable sites (1.07%) with 1,192 (0.74%) being parsimoniously informative (Supplementary Table 3). The ML (Figure 3A) and BI (Figure 3B) analyses of the concatenated plastome matrix produced similar tree topologies except that several shallow nodes with low BS support collapse in BI tree. Two diverging clades were recovered in *P.* sect. *Axiparis*: Clade I (BS = 100%, PP = 1.00) comprises accessions of *P. dulongensis*, *P. forrestii*, *P. rugosa*, and *P. tengchongensis*, and Clade II (BS = 100%, PP = 1.00) includes accessions of *P. axialis*, *P. guizhouensis*, *P. lihengiana*, *P. vaniotii*, and *P. variabilis*. Strikingly, none of the nine nominal species within *P.* sect. *Axiparis* were recovered as a monophyletic unit in either ML or BI phylogeny.

**FIGURE 4:**
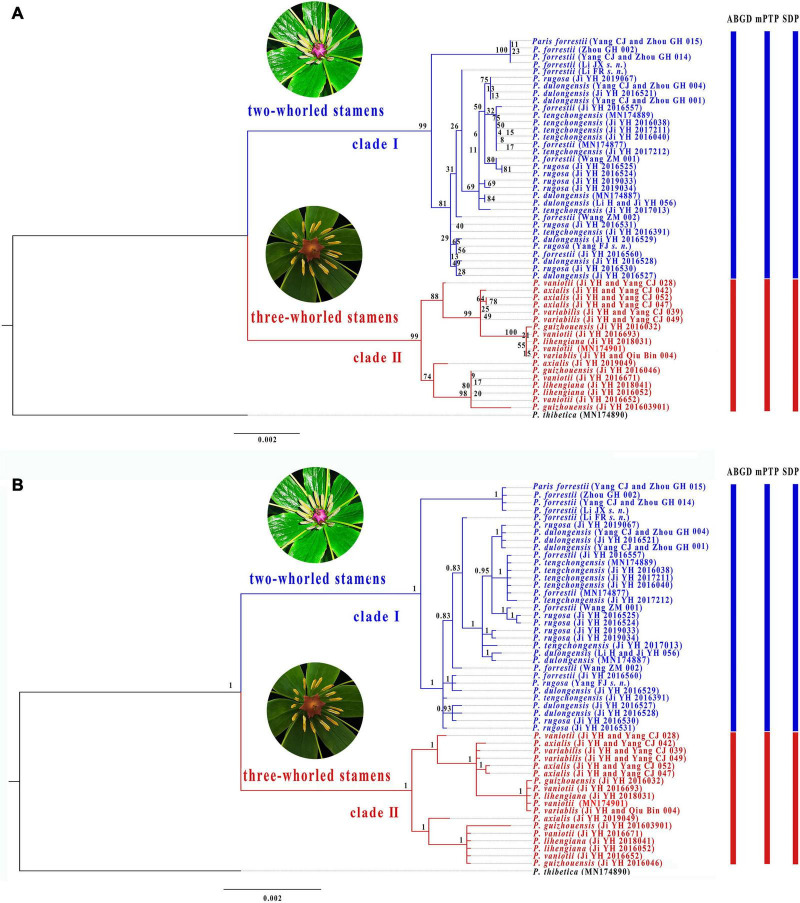
Phylogenetic tree of *Paris* section *Axiparis* based on Maximum likelihood (ML, **A**) and Bayesian inference (BI, **B**) analyses of nuclear ribosomal DNA (nrDNA). Numbers above branches indicate ML bootstrap (BS) percentages and Bayesian posterior probabilities (PP). Species delimitation schemes proposed by automatic barcode gap discovery (ABGD), multi-rate Poisson tree processes model (mPTP), and species delimitation plugin (SDP) of Geneious are reflected on the tree topology.

Although partition homogeneity tests detected significant conflicts (*p* < 0.01) between the nrDNA and plastome phylogenies, the coding (18S rRNA, 5.8S rRNA, and 26S rRNA) and non-coding regions (ITS1 and ITS2) of nrDNA sequences are congruent with each other in the tree topology (*p* > 0.05). Therefore, we concatenated both coding and non-coding regions of nrDNA sequences for phylogenetic and species delimitation analyses. The concatenated nrDNA matrix possessed 193 variable sites (3.30%) with 112 (1.91%) being parsimoniously informative (Supplementary Table 3). ML (Figure 4A) and BI (Figure 4B) analyses generated congruent tree topologies despite that some weakly supported shallow nodes in ML tree collapse in BI phylogeny. The nrDNA trees recovered two major clades within *P.* sect. *Axiparis*, corresponding to those inferred from the plastomes but with slightly lower support values (clade I: BS = 98%, PP = 1.00; clade II: BS = 97%, PP = 1.00). Similar to the plastome phylogeny, analyses of nrDNA sequences failed to resolve any of the nine nominal species within *P.* sect. *Axiparis* as a monophyletic unit.

### Species Delimitation Scenarios

Overall, ABGD, mPTP, and SDP analyses of the concatenated plastome and nrDNA matrices produced highly congruent results that are reflected in the phylogenetic trees (Figures 3, 4). The ABGD analyses of the concatenated plastome (Table 2) and nrDNA (Table 3) matrices resulted in a stable count (*n* = 2) of species division with a range of prior intraspecific values (plastomes: *P* = 0.001000–0.004642; nrDNA: *P* = 0.002000–0.007573) with JC69, K2P, and *P*-distances initial and recursive partitions. One proposed species includes individuals of *P. dulongensis*, *P. forrestii*, *P. rugosa*, and *P. tengchongensis*, while the other comprises individuals of *P. axialis*, *P. guizhouensis*, *P. lihengiana*, *P. vaniotii*, and *P. variabilis* (Figures 3, 4). The mPTP analyses yielded the same delimitation proposal as ABGD: all individuals were grouped into two species-like entities that coincide with the two clades recovered by our phylogenetic analyses (Figures 3, 4), and both received strong posterior support (Table 4). Similarly, the SDP analyses showed that either plastome or nrDNA matrix, excluding the outgroup, comprised two putative species (corresponding to Clade I and Clade II in the inferred phylogenetic tree; Figures 3, 4), which were distinctive with *P*_(RD)_ < 0.05, and significant with *P*_(AB)_ < 10^–5^ (Table 5).

**TABLE 2:** The number of putative species recognized by Automatic Barcode Gap Discovery (ABGD) analyses among 51 complete plastomes using three distance metrics.

Subst. model	X	Partition	Prior intraspecific divergence (P)			
			0.001000	0.001668	0.002783	0.004642
P	1.5	Initial	2	2	2	2
		Recursive	2	2	2	2
JC69	1.5	Initial	2	2	2	2
		Recursive	2	2	2	2
K2P	1.5	Initial	2	2	2	2
		Recursive	2	2	2	2

*X, relative gap width; P, p-distance; JC69, Jukes-Cantor; K2P, Kimura 2-parameter.*

**TABLE 3:** The number of putative species recognized by Automatic Barcode Gap Discovery (ABGD) analyses among 51 nrDNA sequences using three distance metrics.

Subst. model	X	Partition	Prior intraspecific divergence (P)				
			0.002000	0.002790	0.003892	0.005429	0.007573
P	1.5	Initial	2	2	2	2	2
		Recursive	2	2	2	2	2
JC69	1.5	Initial	2	2	2	2	2
		Recursive	2	2	2	2	2
K2P	1.5	Initial	2	2	2		
		Recursive	2	2	2		

*X, relative gap width; P, p-distance; JC69, Jukes-Cantor; K2P, Kimura 2-parameter.*

**TABLE 4:** Posterior delimitation probability of two putative species proposed by mPTP analyses of complete plastomes and nuclear ribosomal DNA (nrDNA) sequences.

Putative species	Posterior delimitation probability	
	Plastomes	nrDNA
Clade I	1.00	1.00
Clade II	1.00	1.00

**TABLE 5:** Species delimitation plugin (SDP) analyses show the distinctiveness [Rodrigo’s *P*_(_*_*R*_*_*D*)_] and significance [Rosenberg’s *P_(AB)_*] of the two clades recovered by phylogenetic analyses of complete plastomes and nuclear ribosomal DNA (nrDNA) as species-level taxonomic units.

Phylogenetic inference	Putative species	Complete plastomes		nrDNA sequences	
		Rodrigo’s *P*_(_*_*R*_*_*D)*_	Rosenberg’s *P*_(AB)_	Rodrigo’s *P*_(_*_*R*_*_*D)*_	Rosenberg’s *P*_(AB)_
BI tree	Clade I	<0.05	5.9 × 10^–16^	<0.05	5.9 × 10^–16^
	Clade II	<0.05	3.2 × 10^–16^	<0.05	3.2 × 10^–16^
ML tree	Clade I	<0.05	9.1 × 10^–16^	<0.05	9.1 × 10^–16^
	Clade II	<0.05	9.1 × 10^–16^	<0.05	9.1 × 10^–16^

## Discussion

The advancement of molecular-based approaches has brought about great progress in species delimitation (Wiley and Mayden, 2000; Sites and Marshall, 2003; De Queiroz, 2007). Concomitantly, a wide variety of species delimitation methods have been developed, especially over the past 20 years (Yang and Rannala, 2010; Ence and Carstens, 2011; Masters et al., 2011; Puillandre et al., 2011; Rannala and Yang, 2013; Zhang et al., 2013). However, no method is perfect and each has specific weaknesses. For instance, ABGD is problematic when species are represented with only a few specimens (Puillandre et al., 2011, 2012). In some cases, mPTP may lead to an overestimation of species numbers (Song et al., 2018; Huang et al., 2020). Given that factors such as recent diversification, radiative speciation, and restricted intraspecific gene flow may result in the absence of reciprocal monophyly among closely related species (Ruhsam et al., 2015; Hollingsworth et al., 2016); SDP may yield biased delimitation schemes since it primarily relies on reciprocal monophyly to explore species boundaries (Masters et al., 2011). Accordingly, multiple methods should be simultaneously employed to develop a robust species delimitation framework, given that using different delimitation approaches allows accommodations for the weaknesses of each approach (Hebert et al., 2004; Fujita et al., 2012; Aguilar et al., 2013; Kekkonen and Hebert, 2014; Mutanen et al., 2015). However, previous studies using a single or few sequence regions have indicated that employing multiple methods on the same dataset always produced incongruent delimitation proposals (Camargo et al., 2012; Mutanen et al., 2015; Jacobs et al., 2018; Jirapatrasilp et al., 2019) likely due to the inadequacies of available genetic information to properly delineate species boundaries (Carstens and Satler, 2013; Jacobs et al., 2018).

High variability of key diagnostic morphological characters makes species delimitation a particularly difficult task, and thus has led to considerable taxonomic confusion in *P.* sect. *Axiparis*. This study aims to critically test our taxonomical proposal that only recognizes two broadly defined species (vs. nine narrowly defined species) in *P.* sect. *Axiparis*. To achieve this goal, we employed genome skimming to recover complete plastomes and nrDNA sequences that contain more evolutionarily informative variation than a single or few sequence regions for phylogenetic and species delimitation analyses. Based on the concatenated plastome and nrDNA matrices, we not only investigated the species-level monophyly of the nine narrowly defined species by sampling multiple individuals per species, but also explored species boundaries using multiple sequence-based species delimitation methods (ABGD, mPTP, and SDP). To determine the robustness of the delimitation proposals generated by ABGD, mPTP, and SDP analyses, we also examined to what extent these methods generate congruent results.

Phylogenetic analyses recovered none of the nine narrowly defined species within *P.* sect. *Axiparis* as a monophyletic unit, suggesting that the genetic differentiation among them is low. By contrast, both plastome and nrDNA phylogenies recovered two well-supported clades that possess fairly distinct morphological traits (Figures 3, 4) and distribution ranges (Figure 2). Although members of *P.* sect. *Axiparis* represent recently diverged entities with their origins estimated no earlier than the late Miocene (Ji et al., 2019b), ABGD, mPTP, and SDP analyses based on different models generated consistent proposals in delineating species boundaries. Briefly, the ABGD analyses (Tables 2, 3) partitioned all the samples into two clusters comprised of individuals having two-whorled and (Clade I) three-whorled stamens (Clade II). This implies that there are likely two distinct species within *P.* sect. *Axiparis* with significant genetic gaps between them (Puillandre et al., 2012). Moreover, the mPTP analyses grouped all accessions into two putative species with high posterior delimitation probability, coinciding with the results found in the ABGD analyses (Figures 3, 4, and Table 4). The putative species boundaries proposed by ABGD and mPTP analyses are further validated by the SDP analyses: with *P*_(_*_*R*_*_*D)*_ value < 0.05 and the *P*_(AB)_ value < 10^–5^ (Table 5), both of the two candidate species can be recognized as evolutionarily distinctive entities (Rosenberg, 2007; Rodrigo et al., 2008). These reciprocally reinforcing results suggest that only two species-level taxonomic units can be recognized in *P*. sect. *Axiparis*, providing robust support to our taxonomic proposal to reduce *P. axialis*, *P. guizhouensis*, *P. lihengiana*, and *P. variabilis* as the synonyms of *P. vaniotii*, and to expand the species boundary of *P. forrestii* to accommodate *P. dulongensis*, *P. rugose*, and *P. tengchongensis*.

Importantly, sequence-based delimitation results can only be considered a hypothesis that needs to be further validated with multiple data types, such as phenotypic and ecological information (Sukumaran and Knowles, 2017). In addition to morphological distinctiveness (two-whorled stamens vs. three-whorled stamens), there is a range of evidence robustly supporting the species delimitation scheme proposed by our data. Within *Paris*, significant conflict between the plastome and nrDNA trees was also detected in a previous study: although the plastome phylogeny recovered *P.* sect. *Axiparis* as a well-supported clade, it was resolved as non-monophyletic by nrDNA phylogeny (Ji et al., 2019b). By comparing differences in plastome and nrDNA tree topologies, ancient hybridization was proposed to have occurred between the common ancestor of *P. luquanensis* and *P. mairei* (*P.* sect. *Euthyra*) and that of *P. dulongensis*, *P. forrestii*, *P. rugose*, and *P. tengchongensis* (Ji et al., 2019b). This implies that the two broadly defined species, namely *P. forrestii s.l.* (syn. *P. dulongensis*, *P. rugose*, and *P. tengchongensis*) and *P. vaniotii s.l.* (syn. *P. axialis*, *P. guizhouensis*, *P. lihengiana*, and *P. variabilis*), originated separately. Also noteworthy, *P. forrestii s.l.* and *P. vaniotii s.l.* possess allopatric geographic distributions, between which there are great climatic differences. From the late Miocene onward, driven by the intensification of the East Asian monsoon in the summer (An et al., 2001), the species range of *P. vaniotii s.l.* has been primarily governed by Pacific monsoon, whereas that of *P. forrestii s.l.* has been mainly affected by Indian monsoon (Li et al., 2008; Yao et al., 2011; Figure 2). The long-term environmental and ecological heterogeneity resulted from climate differences between the two distinct monsoon regimes would block the regional gene flow and boosted vicariance in *Paris* (Ji et al., 2019b). Based on phenology recorded in herbarium specimens and observation of plants cultivated in common gardens, it was found that the flowering of *P. vaniotii s.l.* is approximately 30–40 days earlier than that of *P. forrestii s.l.*, and there is little overlap between their flowering periods (Ji, 2021). This implies that outcrossing between *P. forrestii s.l.* and *P. vaniotii s.l.* is nearly impossible under natural conditions, suggesting that reproductive isolation between them may have been formed under the combined effect of geographic isolation and habitat heterogeneity. Therefore, recognizing *P. forrestii s.l.* and *P. vaniotii s.l.* as distinct species under the unified species concept (De Queiroz, 2007) is reasonable, given that the species boundary proposed by our data reflects the unity of morphological uniqueness, non-overlapping species range, genetic distinctiveness, and reproductive isolation.

## Conclusion

This study represents a guiding practical application of genome skimming for exploring species boundaries in taxonomically perplexing plant taxa, using not only phylogenetic inferences but also multiple sequence-based species delimitation methods. The analyses of concatenated plastome and nrDNA matrices yielded identical schemes in delineating species boundaries in *Paris* sect. *Axiparis*, which are highly congruent with morphological characteristics and geographic distributions. The results robustly support our revision proposal that reduces the number of species in *P.* sect. *Axiparis* from nine to two, and confirm that previous morphology-based taxonomy (Li, 1984, 1992; He, 1990; Ji et al., 2017; Xu et al., 2019; Yang et al., 2019) overemphasized intraspecific and minor morphological differences to establish species, therefore resulted in over-splitting of species in *P.* sect. *Axiparis*.

## Data Availability Statement

The datasets presented in this study can be found in online repositories. All the relevant accession numbers are included in Supplementary Table 1.

## Author Contributions

YJ, J-BY, and T-SY conceived and designed the research framework. YJ, JY, and LJ collected samples. JY, J-BY, SW, LJ, and PX collected and analyzed the data. YJ wrote the original draft manuscript. JL, T-SY, and HL revised and edited the final manuscript. All authors have read and agreed to the published version of the manuscript.

## Conflict of Interest

The authors declare that the research was conducted in the absence of any commercial or financial relationships that could be construed as a potential conflict of interest.

## Publisher’s Note

All claims expressed in this article are solely those of the authors and do not necessarily represent those of their affiliated organizations, or those of the publisher, the editors and the reviewers. Any product that may be evaluated in this article, or claim that may be made by its manufacturer, is not guaranteed or endorsed by the publisher.

## Footnotes

1
http://purl.org/phylo/treebase/phylows/study/TB2:S29422

2 https://bioinfo.mnhn.fr/abi/public/abgd/abgdweb.html; the latest access date: July 9, 2020.

3 http://species.h-its.org/ptp/; the latest access date: August 14, 2020.
