# Supplementary material for: Genome Skimming Contributes to Clarifying Species Limits in Paris Section Axiparis (Melanthiaceae)
Source: Front Plant Sci. 2022 Apr 4;13:832034. doi: 10.3389/fpls.2022.832034 (PMC9014178; doi:10.3389/fpls.2022.832034)
Supplement: Supplementary file 1 [file Table_1.DOCX]

**Table S1.** Summary of genome skimming.

| Taxon | No. of clean reads | Plastome | | | Ribosomal DNA sequences | | | SRA Accession |
| --- | --- | --- | --- | --- | --- | --- | --- | --- |
|  |  | No. of mapped reads | Size of plastid genome (bp) | Coverage (×) | No. of mapped reads | Size of nrDNA (bp) | Coverage (×) |  |
| *Paris axialis* (Ji YH 2019049) | 13,363,710 | 967,500 | 156,643 | 763.218 | 5,286 | 5,852 | 111.295 | SAMN26001754 |
| *P. axialis* (Ji YH and Yang CJ 042) | 19,557,260 | 514,608 | 156,612 | 492.099 | 10,083 | 5,852 | 260.040 | SAMN26001766 |
| *P. axialis* (Ji YH and Yang CJ 047) | 15,104,578 | 370,249 | 156,521 | 354.054 | 11,154 | 5,852 | 287.661 | SAMN26001770 |
| *P. axialis* (Ji YH and Yang CJ 052) | 16,365,354 | 360,814 | 156,658 | 345.032 | 13,019 | 5,852 | 335.759 | SAMN26001769 |
| *P. dulongensis* (Yang CJ and Zhou GH 001) | 14,037,424 | 740,484 | 157,553 | 701.545 | 6,802 | 5,851 | 172.620 | SAMN26001755 |
| *P. dulongensis* (Yang CJ and Zhou GH 004) | 14,468,708 | 313,565 | 157,553 | 295.371 | 8,006 | 5,851 | 203.536 | SAMN26001765 |
| *P. dulongensis* (Ji YH2016521) | 19,172,060 | 538,143 | 157,566 | 514.604 | 11,056 | 5,851 | 285.133 | SAMN26001730 |
| *P. dulongensis* (Ji YH2016527) | 21,029,164 | 859,579 | 157,294 | 821.980 | 7,284 | 5,851 | 187.854 | SAMN26001731 |
| *P. dulongensis* (Ji YH2016528) | 18,055,298 | 86,006 | 157,248 | 82.244 | 27,881 | 5,851 | 719.049 | SAMN26001732 |
| *P. dulongensis* (Ji YH2016529) | 16,146,410 | 267,156 | 157,306 | 255.470 | 20,961 | 5,851 | 540.583 | SAMN26001733 |
| *P. dulongensis* (Li H and Ji YH 056) | 27,825,002 | 448,275 | 157,342 | 428.667 | 66,322 | 5,851 | 1710.439 | SAMN26001746 |
| *P. forrestii* (Wang ZM 001) | 5,785,510 | 631,819 | 157,534 | 569.078 | 3,875 | 5,851 | 92.582 | SAMN26001758 |
| *P. forrestii* (Wang ZM 002) | 11,914,302 | 363,037 | 157,117 | 336.858 | 7,609 | 5,851 | 191.447 | SAMN26001759 |
| *P. forrestii* (Zhou GH 002) | 18,725,092 | 564,065 | 157,348 | 374.991 | 8,671 | 5,851 | 152.044 | SAMN26001760 |
| *P. forrestii* (Zhou GH *s. n.*) | 21,396,738 | 823,597 | 157,198 | 787.572 | 12,032 | 5,851 | 310.304 | SAMN26036512 |
| *P. forrestii* (Yang CJ and Zhou GH 014) | 16,032,122 | 479,104 | 157,341 | 432.994 | 16,471 | 5,851 | 406.737 | SAMN26001761 |
| *P. forrestii* (Yang CJ and Zhou GH 015) | 16,620,374 | 703,873 | 157,349 | 571.033 | 6,789 | 5,851 | 148.057 | SAMN26001763 |
| *P. forrestii* (Li JX *s. n.*) | 19,969,508 | 1,053,966 | 157,339 | 997.118 | 7,756 | 5,851 | 196.045 | SAMN26001764 |
| *P. forrestii* (Ji YH 2016557) | 33,107,554 | 103,347 | 157,643 | 98.827 | 27,629 | 5,851 | 712.550 | SAMN26001736 |
| *P. forrestii* (Ji YH 2016560) | 21,292,884 | 241,940 | 157,255 | 231.357 | 22,297 | 5,851 | 575.038 | SAMN26001737 |
| *P. forrestii* (Li FR *s. n.*) | 27,417,778 | 1,044,267 | 157,038 | 998.590 | 36,404 | 5,851 | 938.856 | SAMN26001739 |
| *P. guizhouensis* (Ji YH 2016032) | 14,520,008 | 872,415 | 156,515 | 834.255 | 6,012 | 5,852 | 155.049 | SAMN26001774 |
| *P. guizhouensis* (Ji YH 201603901) | 22,944,742 | 1,068,260 | 156,094 | 1021.533 | 24,952 | 5,852 | 643.510 | SAMN26001741 |
| *P. guizhouensis* (Ji YH 2016046) | 22,145,944 | 923,183 | 156,124 | 882.802 | 34,927 | 5,852 | 900.765 | SAMN26001742 |
| *P. lihengiana* (Ji YH 2016052) | 31,454,974 | 1,789,428 | 156,280 | 1711.157 | 34,098 | 5,852 | 879.385 | SAMN26001743 |
| *P. lihengiana* (Ji YH 2018031) | 15,621,472 | 956,392 | 156,596 | 914.559 | 6,613 | 5,852 | 170.549 | SAMN26001776 |
| *P. lihengiana* (Ji YH 2018041) | 18,219,242 | 607,217 | 156,370 | 580.657 | 20,818 | 5,852 | 536.895 | SAMN26001745 |
| *P. rugosa* (Ji YH 2016530) | 21,720,026 | 620,895 | 157,218 | 593.736 | 21,619 | 5,851 | 557.552 | SAMN26001734 |
| *P. rugosa* (Ji YH 2016531) | 28,131,814 | 269,604 | 157,311 | 257.811 | 20,121 | 5,851 | 518.919 | SAMN26001735 |
| *P. rugosa* (Ji YH 2016524) | 22,371,000 | 132,045 | 157,113 | 126.269 | 20,527 | 5,851 | 529.390 | SAMN26001738 |
| *P. rugosa* (Ji YH 2019033) | 13,737,170 | 1,123,446 | 157,455 | 1045.053 | 9,601 | 5,851 | 238.065 | SAMN26001756 |
| *P. rugosa* (Ji YH 2019034) | 18,362,956 | 343,133 | 157,508 | 312.407 | 27,289 | 5,851 | 679.477 | SAMN26001757 |
| *P. rugosa* (Ji YH 2019067) | 24,255,172 | 502,092 | 157,499 | 449.419 | 25,716 | 5,851 | 622.929 | SAMN26001762 |
| *P. rugosa* (Yang FJ *s. n.*) | 21,837,302 | 335,002 | 157,168 | 320.349 | 13,934 | 5,851 | 359.357 | SAMN26001752 |
| *P. tengchongensis* (Ji YH 2017211) | 24,767,538 | 263,920 | 157,149 | 252.376 | 12,137 | 5,851 | 313.012 | SAMN26001749 |
| *P. tengchongensis* (Ji YH 2017212) | 19,890,864 | 254,712 | 157,149 | 243.571 | 19,548 | 5,851 | 504.141 | SAMN26001750 |
| *P. tengchongensis* (Ji YH 2016038) | 24,260,456 | 238,707 | 157,149 | 228.266 | 19,050 | 5,851 | 491.298 | SAMN26001747 |
| *P. tengchongensis* (Ji YH 2016040) | 21,577,260 | 267,713 | 157,149 | 256.003 | 24,478 | 5,851 | 631.28 | SAMN26001748 |
| *P. tengchongensis* (Ji YH 2017013) | 29,071,044 | 495,292 | 157,371 | 473.627 | 33,662 | 5,851 | 868.140 | SAMN26001751 |
| *P. tengchongensis* (Ji YH 2016391) | 23,160,682 | 280,033 | 157,152 | 267.784 | 13,292 | 5,851 | 342.800 | SAMN26001753 |
| *P. vaniotii* (Ji YH 2016652) | 23,849,818 | 1,065,822 | 156,266 | 1019.202 | 15,771 | 5,852 | 406.733 | SAMN26001740 |
| *P. vaniotii* (Ji YH 2016671) | 20,134,408 | 521,529 | 156,061 | 498.717 | 32,873 | 5,852 | 847.792 | SAMN26001744 |
| *P. vaniotii* (Ji YH 2016693) | 19,859,162 | 859,926 | 156,578 | 822.312 | 16,335 | 5,852 | 421.278 | SAMN26001773 |
| *P. variablis* (Ji YH and Yang CJ 028) | 15,463,120 | 318,000 | 156,673 | 304.090 | 12,304 | 5,852 | 317.319 | SAMN26001771 |
| *P. variablis* (Ji YH and Yang CJ 039) | 17,228,202 | 346,001 | 156,612 | 330.867 | 13,121 | 5,852 | 338.390 | SAMN26001767 |
| *P. variablis* (Ji YH and Yang CJ 049) | 16,715,604 | 269,449 | 156,697 | 257.663 | 10,136 | 5,852 | 258.487 | SAMN26001768 |
| *P. variablis* (Ji YH and Qiu B 004) | 15,289,022 | 725,946 | 156,578 | 694.192 | 11,491 | 5,852 | 296.352 | SAMN26001772 |
