# Supplementary material for: Genome Skimming Contributes to Clarifying Species Limits in Paris Section Axiparis (Melanthiaceae)
Source: Front Plant Sci. 2022 Apr 4;13:832034. doi: 10.3389/fpls.2022.832034 (PMC9014178; doi:10.3389/fpls.2022.832034)
Supplement: Supplementary file 3 [file Table_3.DOCX]

**Table S3.** Characteristics of complete plastome and nuclear ribosomal DNA datasets in *Paris* section *Axiparis*. Alignment of sequences are deposited in the online database Treebase (http://purl.org/phylo/treebase/phylows/study/TB2:S29338).

| Sequence | Aligned length (bp) | No. of variable sites (divergence %) | No. of parsimony informative sites (divergence %) |
| --- | --- | --- | --- |
| Ribosomal DNA | 5,852 | 193 (3.30) | 112 (1.91) |
| Entire plastid genome | 160,681 | 1,724 (1.07) | 1,192 (0.74) |
